# A feminist phenomenology on the emotional labor and morality of live-in migrant care workers caring for older people in the community

**DOI:** 10.1186/s12877-019-1352-3

**Published:** 2019-11-19

**Authors:** Ken H. M. HO, Vico C. L. CHIANG, Doris LEUNG, Daphne S. K. CHEUNG

**Affiliations:** 1 School of Nursing, Tung Wah College, 31 Wiley Road, Kowloon, Hong Kong SAR; 2 School of Nursing, The Hong Kong Polytechnic University, Hung Hom, Kowloon, Hong Kong SAR

**Keywords:** Emotional labor, Feminist phenomenology, Home care, Migrant care workers, Moral, Culture

## Abstract

**Background:**

Global societal changes, such as increasing longevity and a shortage of family caregivers, have given rise to a popular worldwide trend of employing live-in migrant care workers (MCWs) to provide homecare for older people. However, the emotional labor and morality inherent in their interactions with older people are largely unknown. The aim of the present study is to understand the corporeal experiences of live-in migrant care workers in the delivery of emotional labor as seen in their interactions with older people by: (1) describing the ways by which they manage emotional displays with older people; and (2) exploring their morality as enacted through emotional labor.

**Methods:**

We performed a secondary analysis drawing on feminist phenomenology to thematically analyze data from interviews with 11 female MCWs. Follow-up interviews were conducted with 10 participants. The participants had two to 15 years of experience in caring for older people in their homes in Hong Kong.

**Results:**

Performing emotional labor by suppressing and inducing emotions is morally demanding for live-in MCWs, who experience socio-culturally oppressive relationships. However, developing genuine emotions in their relationships with older people prompted the MCWs to protect the interests of older people. Through demonstrating both fake and genuine emotions, emotional labor was a tactic that live-in MCWs demonstrated to interact morally with older people.

**Conclusions:**

Emotional labor allowed live-in MCWs to avoid conflict with older people, and to further protect their own welfare and that of others. This study highlights the significance of empowering live-in MCWs by training them in ways that will help them to adapt to working conditions where they will encounter diverse customs and older people who will develop an increasing dependence on them. Thus, there is a need to develop culturally appropriate interventions to empower live-in MCWs to deliver emotional labor in a moral manner.

## Background

Emotional labor is the induction or suppression of feelings when there is a dissonance between one’s own inner emotions and the emotions that one is expected to display [1]. It is widely expected in the work of caregiving, as it is crucial to the demonstration of compassion [2]. However, the inappropriate enactment of emotional labor adversely affects patient outcomes [3]. Currently, there is a lack of understanding about the emotional labor of live-in migrant care workers (MCWs) who care for older people at home, and about the quality of the care that they deliver.

Global societal changes, such as low birth rates, increasing longevity, and changing family structures, have led to an increasing global trend of shifting the task of caring for older people in the community from family members to live-in migrant care workers (MCWs) [4–6]. This is happening in places such as Asia [7, 8], Canada [9], and in many European nations [10–13]. The Hong Kong Special Administrative Region (hereafter, Hong Kong) is an affluent but ageing city located in the southern part of China. In Hong Kong, around 182,000 community-dwelling older people are being cared for by live-in MCWs (locally known as foreign domestic helpers) [14]. The number of households comprised of retired couples or single older people who have live-in MCWs has increased by threefold and fourfold from 1995 to 2016, respectively [15]. They perform both caring work and domestic work [16, 17], which are generally described as three Cs: cooking, cleaning and caring [18]. However, the boundary between care work and domestic services is usually blurred [19, 20]. Due to the live-in arrangement, MCWs virtually perform round-the-clock duties and 11% of them do not have any day off [14], which affects their wellbeing. For example, 52% of live-in MCWs experienced inadequate sleep and 73% of them did not have regular meal time [14]. On the other hand, live-in MCWs were found to be consistently responsible for around 9% of older people abuse from 2005 to 2018 [21]. Scholars argued that the live-in arrangement and one-to-one interactions intensified tensions between older people and live-in MCWs [22], which were exacerbated by cultural differences and language barriers [10]. Internationally, the dynamic interactions between this (MCWs-older people) dyad are largely unknown [12, 23].

Emotional labor is an important skill to have in negotiating relationships. It involves the complex deployment and self-management of emotions in interpersonal interactions [24]. However, it has often been characterized as a tacit, uncodified skill that is natural to the disposition of women [25]. This has contributed to causing emotional labor to be taken for granted and its potential economic and emotional consequences to be overlooked [26]. Taking emotional labor for granted devalues the work of caregiving and leaves live-in MCWs vulnerable to emotional over-involvement and psychosocial distress [27].

In a systematic review conducted in China, 50% of live-in workers stated that they found it difficult to manage their negative emotions in interpersonal interactions [7]. Common stressors that trigger their negative emotions included the renewal of their employment contract and abusive behavior by employers, such as physical and verbal abuse, sexual harassment, and economic exploitation [7, 28]. For example, if an employer or a live-in MCW terminates an employment contract in Hong Kong, the live-in MCW has to leave Hong Kong within two weeks if s/he cannot start a new employment contract within those weeks. The two-week period is often a stressor for MCWs who wish to find a new employer [29]. Furthermore, the rights of the employer often trump those of the live-in MCWs [30], which largely attribute to a lack of social support, a lack of legal protection, and gender inequality on live-in MCWs [31]. However, these studies do not explain how live-in MCWs employ emotional labor to interact with older people. While emotional labor plays a role in dyadic relationship negotiations [32], it directly influences the quality of care received by community-dwelling older people with live-in MCWs [33]. Therefore, there is a pressing need to understand the emotional labor enacted by live-in MCWs in caring for older people.

Given that emotions contribute to moral reasoning and moral behavior [34], this study takes the lens of moral economy to understand the emotional labor and the enactment of the morality of live-in MCWs in providing care for older people at home [32]. The moral economy is a system of socially acceptable (i.e., moral) transactions that serve to maintain balanced social relationships, through which social ties are recognized [35]. The highly personalized and private sphere of domestic and caregiving work tends to transform relationships into a moral relationship, on the basis of good/bad notions [32]. For instance, live-in MCWs are working under the dominance of a Chinese masculine culture in Hong Kong, in which individuals are expected to demonstrate behaviors that are supposed to represent “good” or “bad” ways of being, according to a hierarchical relationship [36]. In addition, sharing and expressing emotions imply vulnerability and weakness for Chinese [37]. Through understanding the bodily enactment of emotional labor by live-in MCWs, this study provides contextual insights on how caregiving relationships with older people are negotiated morally in Hong Kong. This is posited to contribute to practice and policy, as well as to comparative studies with countries where live-in MCWs are employed to care for the older people. The 32-item COREQ [38] was used as a guide to assure comprehensive reporting of the study.

## Methods

The aim of this study is to understand the corporeal experiences of live-in MCWs in the delivery of emotional labor by: (i) describing the ways by which they manage emotional displays with older people; and (ii) exploring the morality enacted through the emotional labor of live-in MCWs.

The findings of this paper were drawn on a secondary analysis of the data from a primary study by the first author, which discussed the transitions experienced by live-in MCWs as they shifted from a task-oriented relationship to becoming companions of older people through their care work [39]. Taking the perspective of feminist phenomenology by bringing together hermeneutic phenomenology and feminine ethics [40–42], feminist methodology provides a way of understanding the corporeal experiences of the emotional labor of female live-in MCWs. While hermeneutic phenomenology examines the nature of the self-other (relationality) in a relationship, the lens of feminine ethics reveals how an individual’s morality is experienced through one’s own body in caregiving relationships within masculine power structures [42].

### Participants and sampling

The sampling strategy of this study was purposive that fits the need of selecting information-rich cases related to the phenomenon of interest in qualitative inquiry [43]. Live-in MCWs were eligible to participate in this study if they had a minimum of two years of experience in caring for community-dwelling older people in Hong Kong. MCWs who were unable to speak in English or Cantonese (a dialect in Hong Kong) were ineligible for the study. Non-governmental organizations (NGOs) that provide services to MCWs were briefed about the eligibility of potential participants. Upon referral from NGOs or from personal networks, the first author double-checked the eligibility of the potential participants. Although we tried to maximize the number of participants from different nationalities in our study, we were only able to recruit Filipinos and Indonesian participants because they make up the majority (98%) of live-in MCWs in Hong Kong [44]. The first author provided briefing sessions to each potential participant to explain the study, and also distributed an information sheet to each of them before gaining their written consent to voluntarily participate in this study.

### Data collection

The first author audiotaped unstructured interviews ranging from 60 min to 120 min in duration, and transcribed them verbatim. The live-in MCWs were encouraged to lead the interviews with open-ended questions. Probing questions further explored meanings as part of the participants’ moral agency to engage in emotional labor. Examples of the interview questions are listed in Table 1 [39]. Follow-up interviews were conducted with ten participants to facilitate the research team to develop deeper interpretations on live-in MCWs’ corporeal experiences. Live-in MCWs were presented with a transcript of their own anecdotes and was invited to share their views on our preliminary findings. Examples of follow up interview questions included “Regarding our findings (on a theme), does it make sense to you? Why yes or why not?” or “Please elaborate your comments on our findings”. Notably, none of the participants experienced emotional distress during the interviews.

**Table 1:** Examples of interview questions

Starting questions	
● Please share anything that is important to you in your caring for older people. ● How do you interact with older people every day?	
Probing	
● Regarding (an issue/emotion), can you tell me a specific event? ● How did you justify your actions in that (particular situation)? Please give some examples. ● You have just shared a special moment/feeling with me. I want to know more about it. Please feel free to bring up your ideas. ● What were your considerations/feelings?	
Ending the interview	
● Please feel free to share anything more with me.	

### Ethical considerations

Ethical approval was granted from the Hong Kong Polytechnic University [HSEARS20130804001–01]. Given that live-in MCWs are a marginalized and vulnerable group who provide care to older people, they provided their voluntary informed written consent to participate in this study without going through their employers, care-recipients, or employment agencies. Confidentiality was assured in the dissemination of all the findings through the strict use of pseudonyms, and any distinguishing details of the participants’ identity were removed.

### Data analysis

A thematic analysis focused on the existential of lived body was conducted to enable a multilayered understanding of the phenomenon [45, 46]. One such layer was how a sense of morality progressed in a person’s narratives, following a pattern, and organized within episodes of emotional labor [42]. The analysis was a recurrent process of reflection on the lived body experience through (a) converting data into anecdotes; (b) subjecting the anecdotes to holistic, selective, and line-by-line thematizations; and (c) engaging in phenomenological reflective writing [45], in which the dominant power structure was questioned when viewing the emotional labor of live-in MCWs. Eventually, the data capturing the enactment of morality through the emotional labor of live-in MCWs was synthesized into themes. The analysis of the data from the eleven participants revealed recurrent meanings and patterns; hence, temporal saturation was determined and the data analysis ended.

## Results

Thirteen female live-in MCWs were approached and two of them refused to participate because of no day off. Eleven female live-in MCWs (Filipinos: 7; Indonesians: 4), aged 27–57, with 2–15 years of experience in caring for older people in Hong Kong, were recruited from two local NGOs and personal networks. All the eleven participants completed the interview and one of them did not attend the follow-up interview because of busy schedule. The characteristics of participants are provided in Table 2 [39].

**Table 2:** Demographic characteristics of the live-in MCWs

Pseudonyms	Gender	Age group	Nationality	Years of experience in caring for older people in Hong Kong	Number of older people being cared for
Audrey	F	27–36	Indonesian	2–7	2
Bonita	F	37–46	Filipino	8–13	1
Catherine	F	27–36	Indonesian	2–7	3
Dora	F	27–36	Indonesian	2–7	1
Ella	F	37–46	Indonesian	2–7	2
Felicia	F	47–56	Filipino	2–7	2
Gigi	F	57–66	Filipino	8–13	1
Hera	F	47–56	Filipino	14–19	2
Ida	F	47–56	Filipino	8–13	2
Jessica	F	37–46	Filipino	2–7	1
Katy	F	27–36	Filipino	2–7	1

The participants’ emotional labor was characterized by a main theme and three subthemes. The main theme was: The moral habitus of live-in MCWs in managing relationships with older people. The three subthemes describing the enactment of emotional labor and morality in different contexts were: (i) Caregiving regulated by emotions and its expressions as morally demanded by a socio-culturally oppressive work relationship; (ii) Motivated by genuine emotions in the best interests of older people; and (iii) Demonstrating emotions (fake or genuine) for the sake of good stewardship.

### The moral habitus of live-in MCWs in managing relationships with older people

Moral habitus refers to the development of an individual’s moral judgment of the self and of others, and is founded on a matrix of cognized emotions and embodied cognitions that build up over the course of one’s life [47]. The moral habitus of live-in MCWs was evidenced by the development of a sense of agency in MCW to protect the welfare of the older people in their dyadic relationship; grounded in the MCW’s cognized emotions of love and physical expressions, and feelings of love within their bodily experience:

I just try to permit something to develop. … All of our experiences turned into love. In caring for grandmother, I just let her feel assured. No matter what happened, I helped her. Whenever she cried, I cried with her. Through crying, there was bonding because I understood what she felt. (Bonita)

### Caregiving regulated by emotions and its expressions as morally demanded by a socio-culturally oppressive work relationship

Social norms and the policies of a business contract, intertwined with cultural norms in Hong Kong, reinforce oppressive power structures for the MCWs, as they are directly employed by older people or their family member(s). As mentioned, the two-week period is often an issue for MCWs [29]. Therefore, MCWs feel that they have no choice but to follow the orders of older people, in order to keep their job: *He just said that he was my boss; I had to follow his instructions.* (Ella). As such, MCWs suppressed their emotional display in order to complete their given mission, even under a situation of negative experience:

One Saturday, we were waiting for a taxi to take us to dinner with grandmother’s son. She was annoyed, as is usual whenever she has to wait. I was also disturbed by the long wait. She nagged, “It’s too long … .” I tried to comfort her, “Just wait for a moment”. Suddenly, she slapped me hard across my face. I shouted to her, “What the hell are you doing?” However, I still tried to comfort her again, “Your son wants you to dine with him. Just wait for a moment longer”. My heart was painful. (Audrey)

In addition, the suppression of genuine emotions according to Chinese behavioral norms may make it necessary for some MCWs to display fake emotions. For instance, an MCW expressed the need to apologize to her employer, out of respect for his/her authority (indeed it was a moral stance for the MCW):

For Chinese, it is not good for the helper to cry frequently, right? So I don’t show her why I cry … . After all, I am the one who knows my madam. Therefore, I would say sorry to her. This is because if I did not talk to her, she would not talk to me also. (Jessica)

In order to resolve the emotional dissonance arising from the induction and suppression of emotions, the MCWs actively justified their domestic and care work within extrinsic motives. As such, they regulated their emotions by changing the focus of their thoughts from the burden of their work to the imagined future rewards:

It has to be a happy thing to work. Later on, I will be wealthy. That’s what I have thought about. Therefore, I accept harsh work. There is nothing to be happy for. Later on, I will have more money and I will return home to Indonesia. (Dora)

Under the socio-culturally oppressive relationship, suppressing and inducing emotions were morally acceptable strategies for MCWs in their work with older people, as well as an active strategy to alleviate their own emotional dissonance.

### Motivated by genuine emotions in the best interests of older people

Genuine compassion was demonstrated in the narratives of the participating MCWs, as well as growing sensitivity to the suffering of the recipients of their care, as manifested by endearing references to their older charges, such as “grandmother”:

In fact, she was not capable of doing anything. I tried to help her. In fact, I loved her and I treated her just like my grandmother. In the later stage, she might have been just looking for love from other people. She was actually lacking in love. I wanted to take care of her and she just needed a little assurance. (Bonita)

This genuine compassion helped the MCWs to focus on the welfare of the older people. As such, protecting the best interests of the older people became a moral justification for the MCWs. However, their genuine feelings towards their older charges also induced emotional distress, such as worry and fear, which magnified the moral burden embodied in domestic and care work [34]:

Before I left the home for vacation, I also questioned if the older couple can handle themselves because grandmother used to be sick. I also worried if grandfather deteriorated and was unable to answer the phone. That’s why I always spoke for grandfather if hospital staff called in about his condition. Their children are not in Hong Kong; therefore, I take care of everything. Although their children will not blame me in case of any incidents, I still worry if they are home alone. (Catherine)

Paradoxically, the moral burden suffered by the MCWs vividly highlighted their genuine connection with the older people, which was morally meaningful for MCWs. This enactment of morality alongside the emotion work of the MCWs will be further illustrated.

### Demonstrating emotions (fake or genuine) for the sake of good stewardship

The MCWs utilized their close connections with older people to situate them in a morally advantageous way. Through demonstrating fake emotions, the MCWs induced emotions grounded in what was deemed to being a good steward for older people, from the perspective of MCWs. This demonstrated the nuanced moral relationship of how the MCWs made sense of their emotional labor with moral reasoning, and justified good care work from their perspective:

Because of their health, I want them to follow my instructions so that they can take the correct medicines. They must follow. If they don’t listen to me, I would say, “It’s fine. I leave (meaning quitting the job).” Then they will obey my instructions. (Felicia)

Enjoying asymmetrical reciprocity facilitated the MCWs’ burdens. Asymmetrical *reciprocity* refers to the expression of moral respect arising from one’s varying social positions and life histories [48]. Thereby, being treated humanely by employers, as a moral enactment of self-respect [49], was important for the MCWs to remain as a caregiver, more so than the workload itself:

For me, treating me nicely is important as a human being. I don’t mind working hard and I don’t care about difficulties in working. What I ask for is to treat me as a human being, treat me nicely. (Ida)

## Discussion

While it seems universally understood that domestic care is not merely about working relationships, but is morally-based [32], this study contributes to the exploration of emotional labor as playing a key role for MCWs in protecting the welfare of older people, as well as their own. Workers are conventionally thought of as passive enactors of emotional labor. They are expected to behave appropriately and universally under organizational constraints, and within the boundaries of job autonomy and their supervisor’s support [50]. Yet, this study acknowledges that the transformation of working relationships, though morally based, does not eliminate the oppressive power of the employer-employee relationship. Indeed, our findings focused on moral habitus, to support the assertion that while emotions may be universal to humans, the causes, consequences, and expressions of emotions are heavily shaped by social and cultural contexts [47].

In this study, live-in MCWs demonstrated embodied sensitivity to regulate their emotional expressions according to the inherent power structures of their work within Chinese cultural contexts. This effectively allowed them to avoid conflicts with older people and further protected their own interests (e.g., secure employment), and that of the older people (e.g., a sense of health and well-being). Our findings highlighted the cross-cultural nature of migrant care, where culture is a social factor regulating emotional labor and, indeed, is necessary for the “proper” enactment of morality [24].

Although an emotional component is universal in care work [2], the importance of socializing culturally sensitive moral values and socially acceptable behavior, such as Chinese norms of behavior, was demonstrated by the MCWs in this study. As such, the findings suggest that what is socially acceptable in a specific context contributes to the agency of live-in MCWs. With this nuanced knowledge, and an ability to enact it, an MCW’s performance (or lack thereof) of emotional labor can be interpreted as having consequences.

Ho and Chiang argued that improving cultural competency would facilitate the adaptation of migrant care workers [51]. Scholars also showed that most employers prefer MCWs who share similar values and beliefs as them, because of the ease of communication [52]. Currently, a number of studies have focused on the acculturation needs of healthcare professionals, especially medical graduates in a pediatric specialty [53–55]. The difference in the cultural backgrounds of older people and live-in MCWs may be a major barrier to effective interaction [10]. Our findings highlight the pressing acculturation needs of live-in MCWs to the customs and ways of caring in host countries, which affect the proper enactment of emotional labour. An awareness of these needs is a starting point to helping MCWs learn to express emotions in socially acceptable ways, contributing to the quality of the dyadic relationship with older people.

Moral habitus suggests that moral judgment is preceded by cognized emotions and embodied cognitions, with emotions and cognitions being enacted differently in different contexts [47]. Using feminist phenomenology, we successfully captured the corporeal experience of MCWs to express and feel both “fake and genuine” emotions for moral enactment, such that MCWs demonstrated good stewardship, demonstrating the credibility of this study [56]. The MCWs enacted emotional labor through sensible bodily expressions of fake or genuine emotions according to various interactive contexts with older people as a means to a moral end. As such, our finding further supports the view that morality is grounded on a matrix of bodily, cognitive, and social inputs [47, 57], which demonstrated the confirmability of the findings [56].

As explicated, morality and expressions of emotion are socially and culturally contextualized. The structural power hierarchy needs to be taken into account when designing training for the acculturation of live-in MCWs. For example, Liang [58] discussed how a globalized gendered-racialized ideology was actualized through various structured processes of recruiting and disciplining live-in MCWs in Taiwan. Our finding of moral habitus further suggests that empowering live-in MCWs to develop awareness of their moral causes and consequences upon enacting emotional labor is a niche area in culturally contextualized training, which can allow caring relationships to be built on an oppressive employer-employee relationship. Through the insight of moral habitus, our findings can be applied to other cultures with the significance to empower MCWs to appropriately enact emotional labor in various socio-cultural context, which demonstrated the transferability of this study [56].

Current cultural competency training for graduate healthcare students tends to cover topics of racism/discrimination, different worldviews, cultural identity, and general concepts about culture [59]. Although the training was effective at increasing their knowledge, its effects on improving their cultural attitudes, awareness, and especially skills were inconclusive. This may be due to an over-emphasis on acquiring knowledge rather than the skills to deliver culturally competent services [59]. On the contrary, live-in MCWs are given very little structured training to serve as caregivers, as there is an immediate demand for their services. In accordance with our findings that MCWs were required to socialize customs for negotiating relationships with older people, a survey showed that 49% of live-in MCWs expressed a need for training to manage relationships with older people [14], demonstrating the dependability of this study [56]. As such, the development of evidence-based training programs for live-in MCWs should include training in the skills as well as the knowledge to cope with contextualized emotional labor, which is a complex and necessary part of their everyday inter-personal interactions [24]. Indeed, the findings suggest that empowering live-in MCWs to enact emotional labor is aligned to the moral purpose of providing quality caregiving to protect the welfare of older people and live-in MCWs.

### Limitations

This study is limited in a sense that all the participants were females. While gender is socio-culturally constructed, our findings may not be immediately applicable to male MCWs with various socio-cultural contexts. However, under the global phenomenon of feminization of migrant care work [16], in which 98% of MCWs in Hong Kong are female [44], our study is able to address an increasingly important issue as more MCWs are engaged to care for older people in ageing societies. Methodologically, feminist phenomenology was able to uncover the tactful enactment of agency in response to socio-cultural structures from the perspective of MCWs in this study. Nevertheless, caring is inevitably relational between the MCWs, older people and even the family members [33]. A more comprehensive picture on the emotional labor and morality in migrant homecare can be developed by involving older people and family members in future studies. With the mixed-method approaches to triangulate qualitative findings on emotional labor and morality with quantitative data, development of effective interventions and policies to foster the quality of migrant homecare will be warranted.

## Conclusions

Emotional labor is pervasive in the domestic care work of live-in MCWs for older people. The findings of our study indicate the significance of emotional labor in protecting the welfare of older people, as well as MCWs. Furthermore, strengthening the acculturation of live-in MCWs is important in helping MCWs to morally enact emotional labor in older people homecare. Through the lens of moral economy, the feminist phenomenological findings of moral habitus suggest that bodily controlled emotional labor is, in fact, an embodied skill and knowledge for MCWs to interact morally with older people, which impacts on their quality of home care.

## Data Availability

The datasets used and/or analysed during the current study are available from the corresponding author on reasonable request.

## Abbreviations

MCWs: Migrant care workers
